# Hyperendemic pulmonary tuberculosis in peri-urban areas of Karachi, Pakistan

**DOI:** 10.1186/1471-2458-7-70

**Published:** 2007-05-03

**Authors:** Saeed Akhtar, Franklin White, Rumina Hasan, Shafquat Rozi, Mohammad Younus, Faiza Ahmed, Sara Husain, Bilquis Sana Khan

**Affiliations:** 1 Department of Community Medicine and Behavioral Sciences, Faculty of Medicine, Kuwait University, PO Box 24923, Safat 13110, Kuwait; 2 Department of Community Health Sciences, Aga Khan University Stadium Road, Karachi 74800, Pakistan; 3 Department of Pathology and Microbiology Aga Khan University Stadium Road, Karachi 74800, Pakistan; 4 Pacific Health & Development Sciences, Victoria, British Columbia, Canada V9A 1S1

## Abstract

**Background:**

Currently there are very limited empirical data available on the prevalence of pulmonary tuberculosis among residents of marginalized settings in Pakistan. This study assessed the prevalence of pulmonary tuberculosis through active case detection and evaluated predictors of pulmonary tuberculosis among residents of two peri-urban neighbourhoods of Karachi, Pakistan.

**Methods:**

A cross-sectional study was conducted in two peri-urban neighbourhoods from May 2002 to November 2002. Systematic sampling design was used to select households for inclusion in the study. Consenting subjects aged 15 years or more from selected households were interviewed and, whenever possible, sputum samples were obtained. Sputum samples were subjected to direct microscopy by Ziehl-Neelson method, bacterial culture and antibiotic sensitivity tests.

**Results:**

The prevalence (per 100,000) of pulmonary tuberculosis among the subjects aged 15 years or more, who participated in the study was 329 (95% confidence interval (CI): 195 – 519). The prevalence (per 100,000) of pulmonary tuberculosis adjusted for non-sampling was 438 (95% CI: 282 – 651). Other than cough, none of the other clinical variables was significantly associated with pulmonary tuberculosis status. Analysis of drug sensitivity pattern of 15 strains of *Mycobacterium tuberculosis *revealed that one strain was resistant to isoniazid alone, one to streptomycin alone and one was resistant to isoniazid and streptomycin. The remaining 12 strains were susceptible to all five drugs including streptomycin, isoniazid, rifampicin, ethambutol, and pyrazinamide.

**Conclusion:**

This study of previously undetected tuberculosis cases in an impoverished peri-urban setting reveals the poor operational performance of Pakistan's current approach to tuberculosis control; it also demonstrates a higher prevalence of pulmonary tuberculosis than current national estimates. Public health authorities may wish to augment health education efforts aimed at prompting health-seeking behaviour to facilitate more complete and earlier case detection. Such efforts to improve passive case-finding, if combined with more accessible DOTS infra-structure for treatment of detected cases, may help to diminish the high tuberculosis-related morbidity and mortality in marginalized populations. The economics of implementing a more active approach to case finding in resource-constrained setting also deserve further study.

## Background

Tuberculosis is a chronic infectious disease caused by *Mycobacterium tuberculosis *and one of the leading causes of mortality worldwide [1,2]. Almost one-third of the world population (about 2 billion) is infected with *M. tuberculosis *and during the past decade even industrialized countries have faced a resurgence of tuberculosis [3]. However, 95% of tuberculosis cases and 98% of deaths due to tuberculosis occur in impoverished countries of Asia, Africa and South America [3-6]. Among these regions, Southeast Asia seems to be the most afflicted: 44% of its population is reported to be *M. tuberculosis *infected [3]. Due to inadequacy of disease surveillance in Pakistan, exact data for tuberculosis incidence, prevalence and tuberculosis-related mortality are not available [7]. However, based on an estimated annual cumulative incidence of tuberculosis as 171 cases per 100,000, Pakistan has been ranked among the top 22 developing countries afflicted by the current tuberculosis epidemic [8]. Like other developing countries, in Pakistan high risk groups may include people with human immunodeficiency virus/AIDS, people with diabetes or cancer, the malnourished, those living with someone who has active tuberculosis, poor indigent people, occupants of homeless shelters and present or former prisoners [9].

Karachi, the major economic center of Pakistan is a cosmopolitan city characterized by having very affluent to low-income marginal neighborhoods with little access to the health care system. Residents of low-income neighborhoods also suffer from overcrowding and malnutrition, and therefore are predisposed to developing tuberculosis. Currently there are little empirical data available on the prevalence and/or incidence of pulmonary tuberculosis among the residents of such marginalized settings in Pakistan. The objectives of this study were to assess the prevalence of, and to evaluate predictors of pulmonary tuberculosis among residents of two low-income peri-urban neighbourhoods in Karachi, Pakistan.

## Methods

### Setting and Study population

A cross-sectional study was conducted in two low-income peri-urban neighbourhoods (Rehri Goth and Baba Island) of Karachi selected as a purposive sample from May 2002 to November 2002. Rehri Goth is located at the south-east extremity of Karachi with a total population of 14000 residing in 1700 households. Baba Island is located within Karachi's industrial harbor and has a total population of 7000 residing in 850 households. The predominant occupation of men is fishing whereas women are mainly engaged in household activities. The complete list of households in Rehri Goth was available from the Department of Community Health Sciences, Aga Khan University, compiled during a recent baseline study, whereas a similar list of households in Baba Island was obtained from local community leaders. To estimate an expected prevalence of sputum-smear positive tuberculosis cases of 0.2 [3,8], with 95% confidence level, and relative precision of the estimate as 0.05, we required 6147 subjects of age 15 years or more to address the study objective [10]. To accommodate non-response and absentees from home at the time of survey, the estimated sample size was further inflated by 10% to 6762 subjects. Given the average household size of 8 subjects in both neighbourhoods and 60% of the population aged 15 years or more, we finally aimed to include 7000 subjects from 1570 households in this study. This sample size for households was allocated proportionally to Rehri Goth (n = 1047) and Baba Island (n = 523). To obtain required sample size from Rehri Goth (with sampling fraction 1.6 rounded up as 2) every second household (a one-in-two systematic sample) was included. With the sampling fraction of 2, there were not enough households in Rehri Goth; therefore, it was decided to run a second round of sampling of households that were not included in the first round. All the household members aged 15 years or more were eligible for inclusion in the study. A similar strategy was adopted to enroll the study subjects from Baba Island.

Five female and four male interviewers fluent in spoken and written Urdu, from each community, carried out data collection. All interviewers underwent training sessions on interview process, questionnaire administration and collection of sputum sample. A questionnaire was designed initially in English, translated in Urdu and then back-translated in English to see if the meanings of the contents were preserved. The final version of this pre-designed structured questionnaire in Urdu was used for data collection, comprising two sections. The first section contained questions regarding the characteristics of households, whereas, second section comprised questions for individual study subject. The questionnaire included questions regarding socio-economic aspects, clinical predictors (signs and symptoms), tobacco use, prior history of respiratory problems and use of any health services for respiratory problems. The first section of the questionnaire was completed with the help of head of the household or his representative, whereas the second section was completed for each of the eligible members of the household if available on first visit. If any eligible member of the selected household was absent during the first visit, up to three subsequent visits were made to enroll that member. If all three attempts to enroll the eligible subject failed, no replacement was sought.

### Sputum sample and bacteriologic analysis

We intended to collect three sputum samples from each subject eligible for sampling. The first sputum sample was obtained after interviewing patients identified as having productive cough of two or more weeks. The second and third were overnight sputum samples collected on the next two consecutive mornings. All sputum samples were transferred to the Microbiology Laboratory of Aga Khan University Hospital on the day of their collection and subjected to direct microscopy by the Ziehl-Neelsen method, bacterial culture and antibiotic sensitivity test [11]. All the identified pulmonary tuberculosis cases were referred to government-run directly observed treatment short course (DOTS) program for treatment. Verbal consent was obtained from the study participants or their parents/guardians for those under 16 years of age. This study was approved by the Ethics Review Committee of Aga Khan University.

### Data analysis

Summary statistics for household level variables were generated to characterize the studied households. Frequencies (%) or means (± SD) of characteristics of participating subjects were computed. Prevalence of pulmonary tuberculosis and its 95% confidence interval (CI) was calculated using varying denominators with Epi-Info ver. 6.04 (CDC, USA). Also, we computed the prevalence of pulmonary tuberculosis adjusted for non-response from those who did not provide sputum samples but otherwise were eligible for sputum sampling. We also computed the prevalence of pulmonary tuberculosis by neighbourhood among subjects who provided one or more sputum samples and made comparisons using χ^2 ^- test of homogeneity of proportions. To evaluate the association between pulmonary tuberculosis status and each of the demographic and social factors, χ^2 ^- test of independence was carried out. Prevalence (%) of clinical factors was assessed among subjects, who provided one or more sputum samples. To relate each of these clinical factors with pulmonary tuberculosis status of the subjects, prevalence odds ratios and their 95% confidence intervals were obtained by logistic regression analysis.

## Results

### Characteristics of study population

In this cross-sectional study from May, 2002 to November, 2002, a total of 9773 residents of two peri-urban neighbourhoods in Karachi were contacted. Of 9773 subjects, 6083 (62.2%) were aged 15 years or more; of these 5479 (90%) participated in the screening process for identification of cases of pulmonary tuberculosis. Urdu and Sindhi were the predominantly spoken dialects, the main occupation among men was fishing and females were generally engaged in household work. About 69% of participants had no formal school education and 11% were current smokers. Of 5479 subjects who participated in the study, 2552 (46.6 %) were men. The mean (± SD) age (completed years) for male and female subjects was 35.0 (± 16.5) and 33.5 (± 15.8) respectively. The residents of both neighbourhoods lived in overcrowded homes as 81% houses have only 1–2 rooms (Table 1).

**Table 1:** Characteristics of households included in the study for case detection and evaluation of predictors of pulmonary tuberculosis in two peri-urban neighbourhoods of Karachi, Pakistan, May 2002–November 2002 (n = 1423)

Characteristic	n	(%)	
Number of households in two study sites			
Rehri Goth	843	59.2	
Baba Island	580	40.8	
Main language spoken at household			
Sindhi	883	62.1	
Katchi	523	36.8	
Others	17	1.1	
House ownership			
Owned	1408	99.0	
Others	15	1.0	
Number of rooms in a house			
1–2	1152	81.0	
3–4	236	16.6	
> 4	35	2.4	
Total monthly income (Rs*) in household (n = 1023)			
≤ 5000	900	88.0	
> 5000	123	12.0	
Type of cooking facility			
Natural gas	410	28.8	
Others	1013	71.2	
Electricity supply (n = 1410)			
Yes	1336	94.8	
No	74	5.2	
Wastewater disposal system (1423)			
Proper	464	32.6	
Improper	959	67.4	
Time (minutes) to reach the health facility (mean ± SD)			(22.3 ± 20.3)
Household size (mean ± SD)			(8.4 ± 4.4)
Number of adults in interviewed (mean ± SD)			(4.8 ± 2.6)

*1 US $ = 58 Pakistani rupees

### Subjects with cough and number of sputum samples

**Figure 1:**
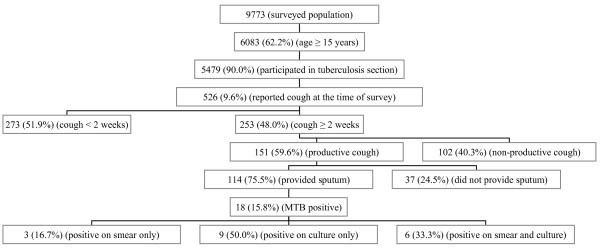
Flow-diagram of a cross-sectional study of pulmonary tuberculosis in two peri-urban neighbourhoods of Karachi, Pakistan; May 2002–November 2002. MTB = *Mycobacterium tuberculosis*

Of 5479 subjects, 526 (9.6%) reported cough at the time of interview, of whom 253 (48 %) reported cough of ≥ 2 weeks of duration. Of 253 subjects with cough, 151 (59.6 %) reported having had a productive cough at the time of study. Of 151 subjects with productive cough, one or more sputum samples were obtained from 114 (75.5 %) respondents (i.e., 1, 2 or 3 samples from 114, 88 and 81 subjects respectively). Sputum samples could not be obtained from 37 (24.5%) subjects who reported having productive cough (Figure 1). The main reasons cited for refusals to provide sputum samples included previously provided samples (for other unrelated investigations) but did not receive the results, forgot to collect sample, or simply refused to provide sample without citing any reason.

### Prevalence of pulmonary tuberculosis

Eighteen of 114 (15.8 %) subjects, who had productive cough of at least 2 weeks duration at the time of interview and provided one or more sputum samples, were identified as suffering from pulmonary tuberculosis. Of these 18 cases, each neighbourhood accounted for 9, reflecting neighbourhood prevalence of 14.3% (9/63) and 17.7% (9/51) in Rehri Goth and Baba Island respectively (*P *= 0.625). Of the 18 cases, 16.7 % (3/18) were positive on smear test alone, 50.0 % (9/18) were positive on bacterial culture alone and 33.3 % (6/18) were positive both on smear and bacterial culture tests. All the 18 cases came from different households, and all provided at least one sputum sample; 14 provided 2 sputum samples and 13 provided three sputum samples. The prevalence (per 100,000) of pulmonary tuberculosis among the subjects aged ≥ 15 years, who participated in the study was 329 (95% CI: 195 – 519) (Table 2). The prevalence (per 100,000) of pulmonary tuberculosis adjusted for non-sampling was 438 (95% CI: 282 – 651).

**Table 2:** Prevalence (per 100,000) (95% confidence interval) of pulmonary tuberculosis in two low-income neighborhoods in Karachi, Pakistan, May 2002–November 2002.

Study population subgroup	Any method (18 positives)	Adjusted for non-sampling of sputum samples (24 positive)
aged ≥ 15 years (n = 6083)	296 (176–467)	395 (253–587)
aged ≥ 15 years, & participated in study (n = 5479)	329 (195–519)	438 (281–651)
All identified with cough (n = 526)	3422 (2041 – 5355)	4563 (2945–6713)
Cough ≥ 2 weeks(n = 253)	7155 (4271 – 11011)	9486 (6173–13786)
Cough ≥ 2 weeks, with ≥ 1 sample (n = 114)	15790 (9635 – 23802)	21053 (13979–29686)
Cough ≥ 2 weeks, with ≥ 1 sample, with blood sputum (n = 32)	56250 (36663–73636)	75000 (56595 – 88538)

Of 114 subjects with cough of ≥ 2-week duration, and provided at least one sputum sample, the results for 102 were in agreement for both positive (6) and negative (96) on smear test and bacterial culture. Sputum samples from 9 subjects were positive on culture and negative on smear, and sputum samples from 3 subjects were positive on smear but negative on bacterial culture. Considering bacterial culture as a gold standard, these results yielded the following parameters' estimates for smear test in these settings: sensitivity 40%; specificity 97 %; positive and negative predictive values 67% and 91% respectively.

Of 18 cases of pulmonary tuberculosis, 11 (61.1%) were male, 9 (50.0%) were aged 45 years or more, 10 (55.6%) reported close contact with a tuberculosis patient and 7 (38.9) reported a prior history of tuberculosis. Apart from male gender (*P *= 0.001), none of the other variables was associated with pulmonary tuberculosis status (Table 3). All 18 cases were referred and treated under DOTS program.

**Table 3:** Comparison of characteristics of study subjects who were having cough of two or more weeks and provided at least one sputum sample for diagnosis of pulmonary tuberculosis (PTB) by smear and/or bacteriological culture test in two peri-urban neighbourhoods of Karachi, Pakistan, May 2002–November 2002; (n = 114).

Variable	PTB Positive	PTB negative	*P *value*
	(n = 18)	(n = 96)	
			
	n (%)	n (%)	
Gender			
Male	11 (61.1)	21 (21.9)	0.001
Female	7 (38.9)	75 (78.1)	
Age (years)			
15–34	6 (33.3)	33 (34.4)	0.965
35–44	3 (16.7)	18 (18.8)	
≥ 45	9 (50.0)	45 (46.9)	
Close contact with TB patient			
Yes	13 (72.2)	46 (47.9)	0.151
No	5 (44.4)	50 (52.1)	
Past history of tuberculosis			
Yes	7 (38.9)	32 (33.3)	0.870
No	11 (61.1)	64 (66.7)	
Current smoking status			
Yes	7 (38.9)	38 (39.6)	0.587
No	11 (61.1)	58 (60.4)	
Current marital status			
Married	10 (55.6)	59 (61.5)	0.498
Single	8 (44.4)	37 (38.5)	
Household size			
≤ 8	13 (72.2)	69 (71.9)	0.611
> 8	5 (27.8)	27 (28.1)	
Education (school years)			
Nil	17 (94.4)	89 (92.7)	0.631
Some	1 (5.6)	7 (7.3)	

*p*-*value *associated with chi-square or fisher exact test statistic in case the expected frequency of any of cell in 2 × 2 table was less than 5.

### Association of clinical variables with pulmonary tuberculosis

The clinical variables evaluated for their relationship with pulmonary tuberculosis status of the subjects included: blood in sputum, night sweat, weight loss, current fever, loss of appetite in previous two months, currently feeling increased fatigue. None of these variables showed statistically significant association with pulmonary tuberculosis status; therefore, multivariate analysis was not carried out (Table 4).

**Table 4:** Predictors of pulmonary tuberculosis (PTB) among subjects identified as having cough and provided one or more sputum samples among residents of two peri-urban neighbourhoods in Karachi, May 2002-November 2002; (n = 114)

Variable	n*	PTB^+^(%)	Crude OR**	95% CI**
Blood in sputum				
Yes	32	5 (15.6)	0.98	0.27 – 3.36
No	82	13 (15.9)	1.00	-
Night sweat				
Yes	43	7 (16.3)	1.06	0.33 – 3.31
No	71	11 (15.5)	1.00	-
Weight loss				
Yes	78	11 (14.1)	0.68	0.22 – 2.18
No	36	7 (19.4)	1.00	-
Presently has fever				
Yes	55	8 (14.6)	0.83	0.27 – 2.55
No	59	10 (17.0)	1.00	-
Loss of appetite in last two months				
Yes	76	12 (15.8)	1.00	0.31 – 3.33
No	38	6 (15.8)	1.00	-
Feeling of increased fatigue				
Yes	92	13 (14.1)	0.56	0.16 – 2.09
No	22	5 (22.7)	1.00	-

* n = total in given category of the variable

** OR = odds ratio; CI = confidence interval

### Drug resistance pattern of M. tuberculosis strains

Analysis of drug sensitivity pattern of 15 strains of *M. tuberculosis *revealed that one strain was resistant to isoniazid alone, one to streptomycin alone and one was resistant to isoniazid and streptomycin. The remaining 12 strains were susceptible to all five drugs including streptomycin, isoniazid, rifampicin, ethambutol, and pyrazinamide.

## Discussion

The poor peri-urban areas of developing countries, wherein living conditions are unsatisfactory with overcrowding, poor hygiene and inadequate sanitation are usually most affected by tuberculosis [12,13]. Such living conditions, lack of access to health-care and/or poor health-seeking behaviour may lead to a vicious cycle of *M. tuberculosis *transmission [13,14]. Furthermore, national notification data often do not reveal the overwhelming burden of tuberculosis in these impoverished populations [15]. We used systematic sampling to select the households from two neighbourhoods for inclusion in our study. As an efficient alternative to a complete census, this is considered a preferred method and has been used for tuberculosis knowledge and attitude assessment in a similar impoverished setting in India [16]

In this study, the prevalence (per 100,000) of pulmonary tuberculosis among those aged 15 years or more, who actually participated in the study was 329 (95% CI: 195–519) and prevalence adjusted for non-sampling was 438 (95% CI: 281–651). These figures appear among the highest in world [8]. It is substantially higher than an earlier reported national figure of 171/100,000 for Pakistan [8]. However, by taking into account the variability of our point estimate of prevalence of 329, the lower limit of the point estimate is not far away from WHO estimates, given the fact that chosen suburbs have higher tuberculosis prevalence than the average figure for Pakistan. Nonetheless, the observed difference could be due to the fact that in the present study we conducted active case detection among those with a cough of 2 or more weeks of duration. By contrast, the WHO figure is an estimate based on certain assumptions regarding Pakistan as a whole, which may not necessarily hold in the overcrowded and impoverished peri-urban settings of this study. Currently, federal or provincial health services do not conduct such active surveillance for pulmonary tuberculosis cases in the study area or elsewhere in the country. Given the confidence intervals for pulmonary tuberculosis prevalence (per 100, 000) of 329 (95% CI: 195–519) and adjusted prevalence of 438 (95% CI: 281–651), our findings are not far off the figure 557/100,000 previously reported in northern area of Pakistan [17]. Alvi et al [17] used a rapid village survey method, which entailed going to every house [18], almost comparable with the sampling design used in this study. Prevalence of pulmonary tuberculosis among those who provided at least one sputum sample was 16.2%, which is twice as high as reported in marginalized communities in Mexico [19]. The factors which possibly could explain this difference may include differing government spending priorities for public health, rates of case detection, and the subsequent treatment of cases which may have reduced the rates of secondary transmission in their settings.

Government health services in Pakistan base their pulmonary tuberculosis case-detection on self-reporting followed by smear testing of suspected cases. Had we followed a similar approach in this study we would not have detected 60% of the present cases at this stage. WHO's proposed target of detecting at least 75% of tuberculosis cases and treatment of 85% of detected tuberculosis cases therefore could not have been met. The greater number of pulmonary tuberculosis cases detected through active case finding as in this study does not exclude the possibility that some of these cases may have been detected at a later stage; however, in the meantime considerable potential for spread of *M. tuberculosis *would exist as a result of such delayed detection. Even with the more active surveillance implemented in this study, the resulting pulmonary tuberculosis prevalence estimates may have been somewhat underestimated since 25% of the subjects eligible for sampling did not provide sputum samples, and only subjects aged 15 years or more were included in the study. Moreover, some of the subjects may have been incubating *M. tuberculosis *infection or perhaps were tuberculosis – positive with non-productive cough.

None of the demographic [20-22], and socio-economic factors [23], was associated with pulmonary tuberculosis status of the subjects in this study. However, there were only 18 cases; the study had very low power to detect any such potential association of interest. Aside from cough of 2 or more weeks duration, which is incorporated within our algorithm, among the other symptoms considered, none was significantly associated with pulmonary tuberculosis status, which is in contrast to the finding of a study in Mexico [19], wherein blood in sputum was significant predictor of pulmonary tuberculosis. This clinical variable was not statistically significant in our study perhaps due to the low power and may merit further evaluation. Taking into account the results of the univariate analysis, we may therefore conclude that only cough of 2 or more weeks of duration seems to be a reliable predictor of pulmonary tuberculosis status in this population.

Of 15 strains tested for their antibiotic sensitivity, only 3 were resistant to streptomycin, indicating relatively low prevalence of Mycobacterial drug resistance in this setting. Also, none of the pulmonary tuberculosis-positive case had history of previous drug treatment.

### Study limitations

In addition to our reliance on sampling, which inevitably will be less comprehensive than a complete census, we could not achieve the desired sample size of households from Rehri Goth, while households from Baba Island were slightly over-represented in our sample. To assess the impact of this potential sampling bias we reexamined socioeconomic status, health-care seeking behaviour and prevalence of pulmonary tuberculosis by stratified analysis and found them to be virtually homogeneous (data not shown); we therefore have no reasons to believe that this sampling bias had any significant influence on our results. The study was designed purposely to identify contagious cases. Diagnosing and treating sputum-smears cases are crucial to a tuberculosis control program. We might have missed some cases by the screening questions (some persons not reporting cough may be sputum positive) or due to lack of sputum samples from subjects positive on screening questions resulting in under-estimation of prevalence of pulmonary tuberculosis. Despite these limitations, our estimate of prevalence is one of the highest in world and other settings in the same region. However, aside from cough, we could not identify other risk factors for pulmonary tuberculosis in this setting; therefore, the determinants of tuberculosis in this community remain unclear. Such additional insight would be invaluable for designing appropriate interventions as well as for generalizability considerations. In the present study all cases came from independent households, therefore, our previously reported set of risk factors for *M. tuberculosis *infection among the contacts of sputum-smear positive tuberculosis in a marginalized population does not seem relevant in this population [24]. Furthermore, a recent study in a marginalized population reported that in high-incidence area like ours, *M. tuberculosis *transmission occurs mainly outside the household [25]. It is important therefore that future studies continue to focus on elucidating the risk factors for community-acquired *M. tuberculosis *infection in this and similar settings.

## Conclusion

This study highlights the poor operational performance of the passive case-detection approach in the current tuberculosis control program and demonstrates higher prevalence of pulmonary tuberculosis than current national estimates. Our results indicate that active detection of cases using the approach outlined in this study and subsequent treatment of cases under DOTS may have the potential to substantially reduce the burden of pulmonary tuberculosis. Since undetected cases continue to spread infection to close contacts, strategies for tuberculosis case detection need to be improved to minimize the *M. tuberculosis *transmission. However, it is also relevant to note that additional costs would arise if active case finding were to be implemented (e.g., we had to screen over five thousand subjects to detect 18 cases); extending such an approach to similar settings in Pakistan and other resource-constrained countries of south Asia would therefore require careful assessment of overall costs and cost-effectiveness [15]. Improving passive case-detection is also a viable option: realizing the shortcomings in current tuberculosis surveillance methods as highlighted by our study, public health authorities at the very least may wish to consider augmenting health education efforts aimed at prompting health-seeking behaviour to facilitate early case detection. Such efforts to improve passive case-finding, if combined with easily accessible DOTS infra-structure for treatment of detected cases, may help to diminish the high tuberculosis-related morbidity and mortality in marginalized populations in Pakistan and other countries of south Asia.

## Competing interests

The authors declare that they have no competing interests.

## Authors' contributions

Conception of the study: SA Proposal writing: SA, FW, RH Coordination and field implementation: SA, SR, MY, FA, SH, BSK Supervision of laboratory work: RH Data management and analysis: SA, SR, BSK Manuscript draft: SA, FW They all approved the final version of the manuscript.

## Pre-publication history

The pre-publication history for this paper can be accessed here:


